# Dried Blood Spots for Viral Load Monitoring in Malawi: Feasible and Effective

**DOI:** 10.1371/journal.pone.0124748

**Published:** 2015-04-21

**Authors:** Sarah E. Rutstein, Mina C. Hosseinipour, Deborah Kamwendo, Alice Soko, Memory Mkandawire, Andrea K. Biddle, William C. Miller, Morris Weinberger, Stephanie B. Wheeler, Abdoulaye Sarr, Sundeep Gupta, Frank Chimbwandira, Reuben Mwenda, Steve Kamiza, Irving Hoffman, Ronald Mataya

**Affiliations:** 1 Department of Health Policy and Management, University of North Carolina at Chapel Hill, Chapel Hill, North Carolina, United States; 2 Department of Medicine, University of North Carolina at Chapel Hill, Chapel Hill, North Carolina, United States; 3 University of North Carolina Project, Lilongwe, Malawi; 4 School of Public Health, Loma Linda University, Loma Linda, California, United States; 5 Department of Epidemiology, University of North Carolina at Chapel Hill, Chapel Hill, North Carolina, United States; 6 Centers for Disease Control, Lilongwe, Malawi; 7 Ministry of Health, Lilongwe, Malawi; 8 College of Medicine, Blantyre, Malawi; Institute of Infection and Global Health, UNITED KINGDOM

## Abstract

**Objectives:**

To evaluate the feasibility and effectiveness of dried blood spots (DBS) use for viral load (VL) monitoring, describing patient outcomes and programmatic challenges that are relevant for DBS implementation in sub-Saharan Africa.

**Methods:**

We recruited adult antiretroviral therapy (ART) patients from five district hospitals in Malawi. Eligibility reflected anticipated Ministry of Health VL monitoring criteria. Testing was conducted at a central laboratory. Virological failure was defined as >5000 copies/ml. Primary outcomes were program feasibility (timely result availability and patient receipt) and effectiveness (second-line therapy initiation).

**Results:**

We enrolled 1,498 participants; 5.9% were failing at baseline. Median time from enrollment to receipt of results was 42 days; 79.6% of participants received results within 3 months. Among participants with confirmed elevated VL, 92.6% initiated second-line therapy; 90.7% were switched within 365 days of VL testing. Nearly one-third (30.8%) of participants with elevated baseline VL had suppressed (<5,000 copies/ml) on confirmatory testing. Median period between enrollment and specimen testing was 23 days. Adjusting for relevant covariates, participants on ART >4 years were more likely to be failing than participants on therapy 1–4 years (RR 1.7, 95% CI 1.0-2.8); older participants were less likely to be failing (RR 0.95, 95% CI 0.92-0.98). There was no difference in likelihood of failure based on clinical symptoms (RR 1.17, 95% CI 0.65-2.11).

**Conclusions:**

DBS for VL monitoring is feasible and effective in real-world clinical settings. Centralized DBS testing may increase access to VL monitoring in remote settings. Programmatic outcomes are encouraging, especially proportion of eligible participants switched to second-line therapy.

## Introduction

Viral load (VL) testing is the preferred method for monitoring antiretroviral therapy (ART) to identify potential adherence problems and treatment failures [1]. Compared to immunological (CD4 cell counts) or clinical staging, VL testing is more sensitive and specific for accurately diagnosing treatment failure, reducing premature or inappropriate switching to second line therapy [2–7]. Delaying treatment changes for patients failing first-line ART increases morbidity and mortality [8, 9] and may lead to accumulation of resistance mutations that compromise second-line ART response [10–13]. With VL monitoring, failing patients are identified sooner [14–18]. Additionally, the avoidance of premature switching prevents the loss of potential life-years on first-line therapy and costs associated with having patients on more expensive and complicated second-line regimens. These concerns are especially relevant in resource-limited settings where third-line options are not widely available.

As recently revised ART guidelines expand treatment eligibility, potentially leading to >20 million HIV infected patients on ART in Africa alone, access to VL monitoring remains poor and identifying appropriate monitoring strategies in resource-limited settings is an urgent global health priority [19, 20]. The benefits of ART, specifically reducing transmission [21] and disease progression [22], are realized only if viral replication is suppressed [23]. Rates of virological failure in sub-Saharan Africa range from 6% to 53%, depending on failure threshold, clinical setting, and ART exposure time [14, 24–31]. Pooled estimates from low- and middle-income countries at 12 months of ART exposure suggest 16% failure [29].

Despite the benefits of VL monitoring, numerous barriers impede scale-up in resource-limited settings. Traditional VL tests used in developed countries are prohibitively expensive and complex for routine use in resource-limited settings because they require laboratory infrastructure for plasma processing, continuous cold-chain, and phlebotomy-trained providers. Point-of-care technologies are under evaluation but are not yet available [32].

The use of dried blood spot (DBS) for specimen collection and subsequent transport to centralized testing laboratories is an appealing alternative to plasma-based VL testing [1, 33–40]. Malawi is one of many countries attempting to incorporate VL monitoring from DBS into ART care [41, 42]. Over 10 years after ART rollout, <1% of Malawian ART patients are on second-line regimens [42], which may reflect providers’ relying primarily on clinical staging criteria to diagnose treatment failure and subsequent under-diagnosis of virological failure.

DBS program feasibility for routine VL monitoring in ART clinics, including timely return of VL results and receipt of confirmatory testing if indicated, has not been assessed outside of controlled studies. Furthermore, the effectiveness of using DBS for VL monitoring in sub-Saharan Africa, specifically if and when eligible patients are switched to second-line therapy, remains unknown. Previous studies have focused on laboratory validation of DBS as compared to plasma for the purposes of identifying virological failure, but have not followed patients through receipt of results and referral for second-line therapy [34, 36, 43, 44]. Evaluations of programmatic effectiveness and feasibility are essential to guide widespread implementation of DBS for VL monitoring. In coordination with the Malawi Ministry of Health (MOH), we conducted a prospective, non-randomized evaluation of DBS for VL monitoring among ART patients managed at districts hospitals in Malawi. Our objective was to evaluate the feasibility and effectiveness of DBS use for VL monitoring, describing patient outcomes and programmatic challenges that are relevant for DBS implementation in sub-Saharan Africa.

## Methods

### Study population

We enrolled adult (≥18 years) patients from five ART clinics in central and southern Malawi. Inclusion and exclusion criteria mirrored MOH eligibility criteria for routine VL monitoring and monitoring based on suspected clinical failure [45]. Patients were eligible for VL testing if they were on first-line ART for 6 months, 24 months, or any 24-month period (+/- 3 months) thereafter (routine monitoring). Patients who did not meet routine monitoring criteria were eligible if they were on first-line therapy ≥6 months and showed signs of clinical failure (World Health Organization [WHO] Stage 3 or 4). Patients were excluded if currently hospitalized, imprisoned, or involuntarily incarcerated in a medical facility.

### Site selection and enrollment

ART clinics within district hospitals were selected based on the size of their retained ART patient population and willingness to both train providers and enroll participants. We validated DBS vs. plasma VL at the two sites with adequate capacity for plasma-processing. During this validation period, all participants provided a venous and fingerstick sample from which a plasma sample, venous DBS (vDBS), and fingerstick DBS (fsDBS) were produced. Interim analyses demonstrated acceptable agreement between plasma, vDBS, and fsDBS [33] and participants enrolled at the final three sites received fsDBS only.

### Sample collection and transport

Sample collection and virological testing methods are presented elsewhere [33]. Briefly, sites were provided with pre-packed kits containing: DBS card, capillary tubes, gloves, sterile lancet, alcohol swab, plastic zip bag, and desiccant. All specimens were collected by ART clinic or hospital laboratory staff. Once dried, cards were transferred to individual zip bags with desiccant sachets and stored at room temperature.

DBS specimens were transported at ambient temperature to the central laboratory in Lilongwe (4–6 hours away) approximately weekly using existing hospital-based vehicles or shipped via specimen shipment service.

### VL testing and result return

Specimens were tested using the Abbott RealTime HIV-1 Assay (Abbott Laboratories, Chicago, IL) (reportable range of 40 to 10,000,000 copies/ml for plasma and lower limit of detection of 550 copies/ml for DBS) at an internationally monitored research laboratory.

Results were returned to clinics using e-mail, short message service (SMS), or phone. Hard-copies of results were delivered via hospital vehicles returning to the clinic or by study coordinators during routine (approximately weekly) site visits. Providers delivered results to participants during scheduled clinic visits.

### Data collection

All activities were conducted by non-study ART clinic personnel. ART staff members were trained in identifying eligible participants, obtaining consent, specimen collection, study sensitization, adherence counseling, and case report form (CRF) completion. We collected participant demographics, clinical history, and ART adherence data. ART history, including date of diagnosis, ART initiation, and reason for initiation, was abstracted from patient clinic records.

### Study visits

Participants were asked to return for VL results one month after enrollment. Participants with elevated VLs (>5,000 copies/ml) received adherence counseling and were instructed to return after two months for a confirmatory draw. Participants who returned for confirmatory draws were told to return within one month for results. Providers were instructed to refer patients with two elevated VLs for second-line therapy.

### Treatment failure definition

Per 2011 MOH guidelines, virological failure was defined as having two sequential VLs >5,000 copies/ml [45]. For patients with plasma results available (validation period), plasma results were used to guide treatment decisions. If vDBS and fsDBS were available, vDBS results were used; fsDBS was used for treatment decisions in all other cases. Laboratory validation of DBS compared to plasma are not discussed further in this paper [33].

### Programmatic Outcomes

Primary outcomes were feasibility and effectiveness of DBS for VL monitoring. Feasibility was measured by: proportion of participants receiving VL results within 3 months of enrollment; laboratory testing turnaround time; delayed result return due to lab delays; delayed result return due to providers failing to deliver available results; proportion of participants with baseline elevated VL receiving confirmatory DBS; time from participant receipt of results to collection of confirmatory specimen; and time from enrollment to second-line treatment initiation among eligible participants. Participants were terminated from the study if results were not delivered ≥6 months of enrollment. Due to staffing constraints, we were not able to assess the proportion of eligible ART patients visiting the clinics who were enrolled.

Effectiveness of DBS for VL monitoring was proportion of eligible (failing) participants who initiated second-line therapy within 12 months (365 days) of enrollment. We also evaluated the proportion of participants who resuppressed (≤5000 copies/ml on confirmatory specimen) as a secondary outcome, suggesting effectiveness of VL monitoring in general, as related to provider-initiated adherence counseling and patient behavior change.

### Statistical methods

We used student’s t-tests (continuous variables) and Pearson's χ^2^ or Fisher’s exact test (categorical variables) to identify demographics and clinical characteristics associated with VL failure and resuppression (≤5000 copies/ml) [46]. We used generalized linear models with a log link and binomial distribution to explore the relationship between time on ART and VL failure (>5000 copies/ml) at enrollment. Factors considered included age, sex, WHO clinical stage at ART initiation, body mass index (BMI), ART regimen, self-reported adherence, and clinical symptoms. We used likelihood ratio (LR) tests to decide which variables to include. We tested interactions between time on ART and symptoms at enrollment to asses if the effect of ART exposure on likelihood of treatment failure was different for participants who showed signs of clinical failure. We evaluated agreement of time on ART (clinic records versus CRFs) using kappa statistics. We conducted a post-hoc sub-group analysis exploring the relationship between CD4 cell count at ART initiation and treatment failure as this may be an important predictor of virological failure [24, 47].

All analyses were performed using Stata (version 13.0; StataCorp, College Station, TX). P-values <0.05 were considered significant.

### Ethical approval

The National Health Sciences Research Committee of Malawi, the Centers for Disease Control and Prevention Ethics Review, and the Biomedical Institutional Review Board at University of North Carolina, Chapel Hill approved this study. All participants provided written informed consent.

## Results

### Study population

**Fig 1:**
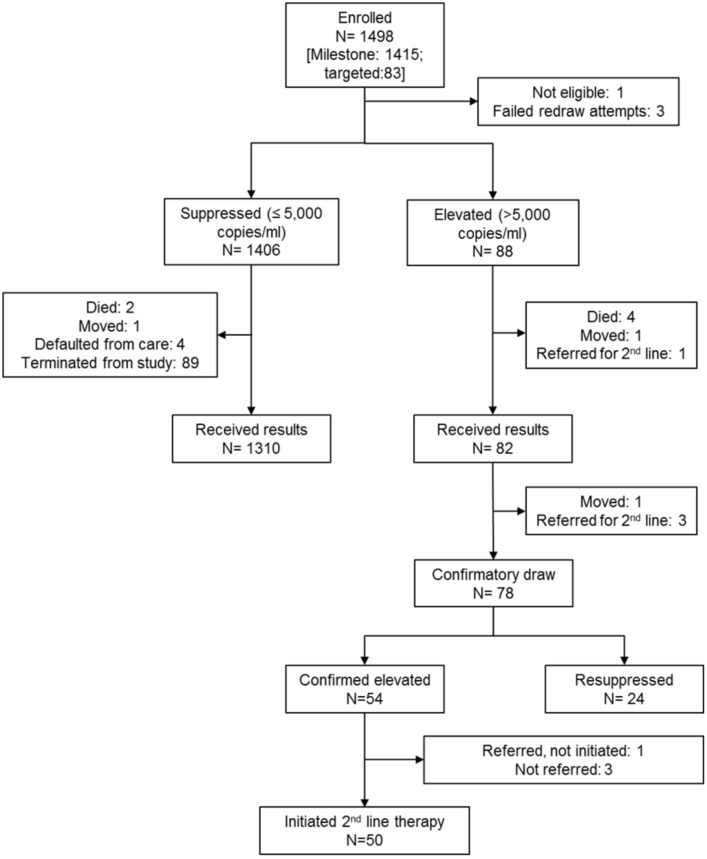
Flow diagram of study enrollment and follow-up. Patients enrolled and eligible for study participation had viral load tests run at a central laboratory. Results were communicated back to enrolling ART clinics where providers were instructed to deliver results to participants. Providers proceeded with clinical care according to if the result was suppressed (≤5,000 copies/ml) or elevated (>5,000 copies/ml). Patients with elevated viral loads received confirmatory testing. Per national guidelines, patients with confirmed elevated viral loads were eligible for second-line ART.

Of 1,498 ART patients enrolled, 1494 (99.7%) had VL results available (Fig 1). The average age was 42.1 years, 444 (29.7%) were male, and most participants had been on ART for ≥2 years (Table 1). Eighty-three (5.5%) were enrolled due to clinical failure. Approximately one quarter (338, 22.8%) had at least one clinical symptom. Only 524 (35.0%) had a quantitative CD4 recorded when initiating ART (mean 187 cells/mm^3^). Nearly three-quarters (1,067, 71.3%) of participants reported 100% adherence over the last 30 days and 1,261 (84.5%) reported 100% adherence over the last week. Pill count was available for 229 (15.3%) participants (99.2% adherent).

**Table 1:** Participant baseline demographics, ART history, & clinical characteristics.

	*All (n = 1*,*494)* ^ᶲ^ N (%)	Suppressed* ≤5,000 copies/ml (n = 1,406) N (%)	Elevated* >5,000 copies/ml (n = 88) N (%)	*p-value*
Participant demographics				
Age (years)				<0.01
18–24	38 (2.5)	31 (2.2)	7 (8.0)	
25–34	323 (21.6)	290 (20.6)	33 (37.5)	
35–44	576 (38.6)	548 (39.0)	28 (31.8)	
45–54	363 (24.3)	350 (24.9)	13 (14.8)	
55–64	158 (10.6)	152 (10.8)	6 (6.8)	
≥65	36 (2.4)	35 (2.5)	1 (1.1)	
Sex				0.61
Male	444 (29.7)	420 (29.9)	24 (27.3)	
Female	1050 (70.3)	986 (70.1)	64 (72.7)	
ART history				
Time on ART (CRF)^†^				0.12
6 months	140 (9.7)	135 (10.0)	5 (5.9)	
2 years	481 (33.3)	453 (33.4)	28 (32.9)	
4 years	340 (23.6)	321 (23.7)	16 (18.8)	
> 4 years**	402 (27.8)	374 (27.6)	27 (31.8)	
Clinical stage at initiation				0.33
Stage 1	213 (16.6)	204 (16.9)	9 (12.5)	
Stage 2	193 (15.0)	177 (14.6)	16 (22.2)	
Stage 3	775 (60.3)	730 (60.3)	41 (56.9)	
Stage 4	105 (8.2)	99 (8.2)	6 (8.3)	
ART regimen				0.36
d4T/3TC/NVP (1A)	835 (55.9)	786 (56.0)	46 (52.9)	
AZT/3TC/NVP (2A)	79 (5.3)	76 (5.4)	3 (3.5)	
TDF/3TC/EFV (5A)	541 (36.2)	505 (36.0)	35 (40.2)	
Other	38 (2.5)	36 (2.6)	2 (2.3)	
Adherence				
No missed doses last 30 days (self-report)	1,067 (71.3)	1003 (71.3)	60 (69.0)	0.64
No missed doses last week (self-report)	1,261 (84.5)	1,189 (84.8)	68 (78.2)	0.10
Clinical Characteristics				
Any symptoms of clinical failure °	338 (22.8)	315 (22.6)	22 (25.3)	0.56
>1 symptom	92 (6.2)	-	-	
>2 symptoms	31 (2.1)	-	-	
Targeted VL monitoring eligibility	83 (5.5)	73 (5.2)	10 (11.4)	0.01
Virological failure (baseline)	88 (5.9)	-	-	
Viral load copies/ml^‡^				
≤5,000	1406 (94.1)	1406 (100.0)	0 (0.0)	
5,000–10,000	11 (0.7)	-	11 (12.5)	
10,001–100,000	54 (3.6)	-	54 (61.4)	
100,001–1,000,000	21 (1.4)	-	21 (23.9)	
≥1,000,000	2 (0.1)	-	2 (2.3)	

^ᶲ^ 1,498 participants enrolled, 1,494 with VL results available.

* “suppressed” and “elevated” refers to baseline viral load measurement as below or above the failure threshold of 5,000 copies/ml;

^†^ Time on therapy collected on study CRFs but only available for patients enrolled under routine monitoring eligibility. ART time was abstracted from clinic records for all enrolled participants.

**267 (59%) on ART for 6 years, 5 (1.1%) for 7 years, 174 (38.6%) for 8 years, and 5 (1.1%) for 10 years.

° Symptoms included: Herpes Zoster, popular pruritic eruption, unexplained chronic diarrhea (>1 month), unexplained persistent fever, moderate unexplained weight loss, oral candidiasis, esophageal candidiasis, pulmonary TB, extra-pulmonary TB, pneumonia, Crytpococcal meningitis, Kaposi’s Sarcoma, and Other.

^‡^ Viral load values assigned as midpoint between 0 and lower limit of detection (40 copies/ml for plasma, 550 copies/ml for DBS). Reported values based on per protocol assessment (plasma or vDBS if available). Median and IQR unchanged among suppressed group if using fsDBS only. Median [IQR] based on fsDBS among patients with elevated VL per fsDBS results was 30,870 [17,156–121,306].

3TC—Lamivudine; ART—antiretroviral therapy; AZT—Zidovudine; CRF—case report form; d4T —Stavudine; DBS—dried blood spot; EFV—Efavirenz; IQR—interquartile range; NVP—Nevirapine; SD—standard deviation; TB—tuberculosis; TDF—Tenofovir; VL—viral load.

### Baseline virological failure

Nearly all participants (1,406, 94.1%) were virologically suppressed at baseline (≤5000 copies/ml) (Fig 1). Compared to participants with suppressed VLs, participants with elevated VLs were younger (37.3 vs 42.4, p<0.01) (Table 1). Sex, BMI, self-reported perfect adherence within the past 30-days or week, and proportion with clinical symptoms were similar according to suppression status. Median VL among participants failing at baseline was 30,329 copies/ml [IQR: 16,483–102,029].

Among 83 persons enrolled for symptoms of clinical failure, 10 (12.1%) had elevated baseline VLs, compared to 78 (5.5%) of persons enrolled based on routine monitoring eligibility (p = 0.01) (Table 2). Participants with clinical failure were older (45.1 vs 41.9, p = 0.01), and more likely to be male (41.0% vs 29.0%, p = 0.02), have advanced WHO stage at ART initiation (p<0.01), and report no missed doses in the last 30 days (81.7% vs 70.7%, p = 0.03). Nearly half of participants enrolled with clinical failure had multiple symptoms (48.2% vs 3.7% routine, p<0.01). Median VL for participants with clinical failure and participants enrolled under routine monitoring eligibility was 275 copies/ml, but the distributions (10^th^ to 90^th^ percentile) differed significantly: routine (275–1,247 copies/ml) versus clinical failure (275–20,150) (Mann-Whitney z = -2.98, p<0.01 two-tailed).

**Table 2:** Routine versus clinical failure participant demographics, ART history, & clinical characteristics.

	*Monitoring*		*p-value*
	*Routine (n = 1*,*415)* N (%)	*Clinical failure (n = 83)* N (%)	
Participant demographics			
Age (years)			0.01
18–24	36 (2.6)	2 (2.4)	
25–34	318 (22.5)	5 (6.0)	
35–44	543 (38.5)	33 (39.8)	
45–54	337 (23.9)	26 (31.3)	
55–64	143 (10.1)	15 (18.1)	
≥65	34 (2.4)	2 (2.4)	
Sex			0.02
Male	410 (29.1)	34 (41.0)	
Female	1001 (70.9)	49 (59.0)	
Time on ART (clinic records)^†^			<0.01
≤1 years	139 (9.9)	6 (7.3)	
1–2 years	460 (32.6)	7 (8.5)	
2–4 years	340 (24.1)	12 (14.6)	
4–6 years	263 (18.6)	26 (31.7)	
>6 years	209 (14.8)	31 (37.8)	
Clinical stage at initiation			<0.01
Stage 1	210 (17.3)	3 (4.0)	
Stage 2	189 (15.6)	4 (5.3)	
Stage 3	715 (59.0)	61 (80.3)	
Stage 4	97 (8.0)	8 (10.5)	
ART regimen			<0.01
d4T/3TC/NVP	806 (57.0)	29 (35.4)	
AZT/3TC/NVP	75 (5.3)	4 (4.9)	
TDF/3TC/EFV	494 (35.0)	47 (57.3)	
Other	37 (2.6)	1 (1.2)	
Adherence			
No missed doses last 30 days (self-report)	1,000 (70.7)	67 (81.7)	0.03
No missed doses last week (self-report)	1,187 (84.1)	74 (90.2)	0.14
Clinical Characteristics			
Any symptoms of clinical failure °	258 (18.4)	80 (97.6)	<0.01
>1 symptom	52 (3.7)	40 (48.2)	<0.01
>2 symptoms	19 (1.4)	12 (14.5)	<0.01
Virological failure (baseline)	78 (5.5)	10 (12.1)	0.01
Viral load copies/ml^‡^			<0.01
≤5,000	1333 (94.5)	73 (88.0)	
5,000–10,000	10 (0.7)	1 (1.2)	
10,001–100,000	52 (3.7)	2 (2.4)	
100,001–1,000,000	15 (1.1)	6 (7.2)	
≥1,000,000	1 (0.1)	1 (1.2)	

^†^ Time on therapy collected on study CRFs but only available for patients enrolled under routine monitoring eligibility. ART time was abstracted from clinic records for all enrolled participants.

° Symptoms included: Herpes Zoster, popular pruritic eruption, unexplained chronic diarrhea (>1 month), unexplained persistent fever, moderate unexplained weight loss, oral candidiasis, esophageal candidiasis, pulmonary TB, extra-pulmonary TB, pneumonia, Crytpococcal meningitis, Kaposi’s Sarcoma, and Other.

^‡^ Reported values based on per protocol assessment (plasma or vDBS if available). Median and IQR unchanged among suppressed group if using fsDBS only. Median [IQR] based on fsDBS among patients with elevated VL per fsDBS results was 30,870 [17,156–121,306].

3TC—Lamivudine; ART—antiretroviral therapy; AZT—Zidovudine; CRF—case report form; d4T —Stavudine; DBS—dried blood spot; EFV—Efavirenz; IQR—interquartile range; NVP—Nevirapine; SD—standard deviation; TB—tuberculosis; TDF—Tenofovir; VL—viral load.

### Predictors of baseline failure

After adjusting for time on therapy, clinical symptoms, sex, WHO stage at initiation, and self-reported adherence, increasing age was associated with decreased risk of failure (RR 0.95, 95% confidence interval (CI) 0.92–0.98) (Table 3). Participants on ART >4 years were 1.7 times more likely to fail compared to participants on therapy 1–4 years (RR 1.70, 95% CI 1.01–2.84); participants on ART ≤1 year were less likely to be failing (RR 0.57, 95% CI 0.18–1.83), although the association was not statistically significant.

**Table 3:** Factors associated with baseline virological failure (>5,000 copies/ml).

Variable	Unadjusted RR (95% CI)	Adjusted RR (95% CI)
Time on ART		
≤1 year	0.81 (0.38–1.71)	0.57 (0.18–1.83)
1–4 years	0.90 (0.60–1.35)	1.0
>4 years	1.21 (0.80–1.82)	1.70 (1.01–2.84)
Age (per year increase)	0.95 (0.93–0.97)	0.95 (0.92–0.98)
Sex		
Male	0.89 (0.56–1.40)	1.42 (0.85–2.36)
Female	1.13 (0.71–1.78)	1.0
Any clinical symptoms at enrollment (yes)	1.15 (0.72–1.83)	1.17 (0.65–2.11)
WHO stage 3 or 4 at ART initiation (yes)	0.87 (0.54–1.40)	0.77 (0.46–1.29)
Self-reported 100% adherence in last 30 days	0.90 (0.58–1.40)	1.13 (0.68–1.89)
Eligible based on targeted monitoring criteria	2.20 (1.17–4.05)	1.54 (0.63–3.77)
BMI (kg/m^2^)	0.99 (0.93–1.06)	N/a

ART, antiretroviral therapy; BMI, body mass index; CI, confidence interval; RR, risk ratio; WHO, World Health Organization.

The effect of time on ART on likelihood of treatment failure did not differ meaningfully among patients with and without documented symptoms of clinical failure (p>0.05). Removing this interaction term did not change model fit (LR test p = 0.26).

Limiting analyses to participants with CD4 count, compared to participants with a CD4 count >100 cells/mm^3^ at ART initiation, participants with CD4 ≤100 cells/mm^3^ were 2.2 times more likely to have an elevated VL after adjusting for time on therapy, age, sex, symptoms, adherence, and BMI (RR 2.22, 95% CI 1.02–4.84). Increasing age remained associated with decreased risk of failure (RR 0.93, 95%CI 0.89–0.98). However, being on ART >4 years was no longer associated with increased risk of failure (RR 1.18, 95% CI 0.50–2.82).

### Resuppression

Among 78 persons with a confirmatory VL, 24 (30.8%) resuppressed (Fig 1, Table 4). Compared to participants who did not resuppress, participants who resuppressed had longer periods between receipt of baseline results and confirmatory testing (median 81.5 vs 60 days, p<0.01). Participants who resuppressed has lower baseline VL than those who did not resuppress (23,167 copies/ml vs 32,562 copies/ml, p = 0.06). Rates of resuppression varied by enrolling clinic (16.7%-56.3%) but differences were not significant according to Fisher’s exact test (p = 0.13).

**Table 4:** Demographic, ART, and clinical outcomes among patients with baseline viral loads >5000 copies/ml.

	*Resuppress (n = 24)* ^†^ N (%)	*No resuppression (n = 54)* ^†^ N (%)	*p-value*
Baseline Characteristics			
Age (years)			0.69
18–24	2 (8.3)	5 (9.3)	
25–34	8 (33.3)	21 (38.9)	
35–44	7 (29.2)	16 (29.6)	
45–54	5 (20.8)	8 (14.8)	
55–64	1 (4.2)	4 (7.4)	
≥65	1 (4.2)	0 (0.0)	
Sex			0.55
Male	7 (29.2)	15 (27.8)	
Female	17 (70.8)	39 (72.2)	
Time on treatment			0.43
6 months	2 (8.7)	2 (3.8)	
2 years	10 (43.5)	18 (34.0)	
4 years	5 (21.7)	11 (20.8)	
> 4 years	4 (17.4)	20 (37.7)	
Enrollment Site			0.13
Site 1	9 (56.3)	7 (43.7)	
Site 2	3 (27.3)	8 (72.7)	
Site 3	4 (33.3)	8 (66.7)	
Site 4	4 (16.7)	20 (83.3)	
Site 5	4 (26.7)	11 (73.3)	
Correct understanding of VL and adherence^‡^	21 (87.5)	50 (92.6)	0.47
Time between baseline and confirmatory VL (days)			0.27
≤90	2 (8.3)	14 (25.9)	
91–180	19 (79.2)	34 (63.0)	
181–270	3 (12.5)	5 (9.3)	
>270	0 (0.0)	1 (1.9)	
Viral load (baseline) copies/ml*			0.35
≤5,000	0 (0.0)	0 (0.0)	
5,000–10,000	4 (16.7)	7 (13.0)	
10,001–100,000	18 (75.0)	33 (61.1)	
100,001–1,000,000	2 (8.3)	13 (24.1)	
≥1,000,000	0 (0.0)	1 (1.9)	

^†^ Among patients with elevated VL, 10 were terminated prior to confirmatory testing; 4 died, 2 moved from the enrolling clinic, and 4 were referred immediately for second-line therapy. Among these 10 terminated patients, 4 were from the routine monitoring group and 6 from the targeted VL group.

^‡^ Question: For most people, if you take all of your medications your viral load will: go up/be higher (*correct*) or go down/be lower (*incorrect*).

*viral load (VL) values assigned as midpoint between 0 and lower limit of detection (40 copies/ml for plasma, 550 copies/ml for DBS).

3TC—Lamivudine; ART—antiretroviral therapy; AZT—Zidovudine; d4T —Stavudine; DBS—dried blood spot; EFV—Efavirenz; IQR—interquartile range; LPV/r—lopinavir/ritonavir; NVP—Nevirapine; SD—standard deviation; TDF—Tenofovir; VL—viral load.

### Programmatic Outcomes

#### Feasibility

Median period between enrollment and a specimen being tested at the central lab was 23 days. Results were communicated back to clinics within 3 days of testing. About 80% (1189/1498) of participants received results within 3 months (mean time to receipt of results 58 days). Nearly 45% (665/1498) of participants had a clinic visit during which VL results were not delivered. Lab-based delays, in which a participant came to the clinic but results were not available, accounted for most delays. However, 21.0% of participants (315/1498) had at least one visit at which results were available, but not delivered.

**Fig 2:**
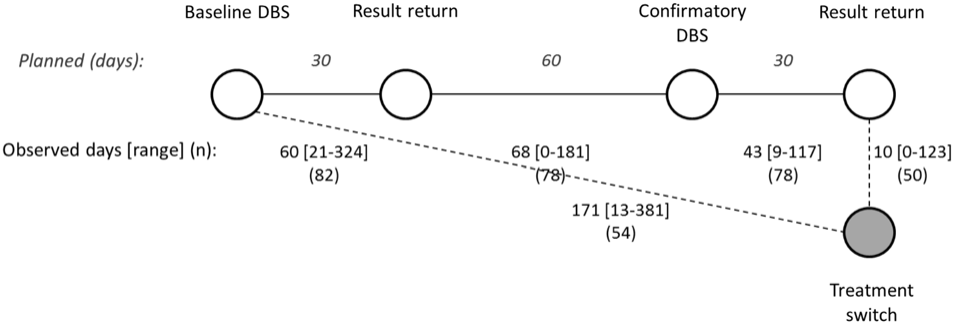
Planned (*italics)* and observed progression through study activities and follow-up for participants with elevated VL at baseline. According to the study protocol, participants were supposed to return for results 30 days after dried blood spot (DBS) collection (baseline DBS). For participants with an elevated viral load (>5,000 copies/ml), confirmatory test specimens were to be collected an additional 60 days later (90 days after enrollment). Again, participants were to return 30 days after DBS collection for receipt of results. This diagram describes the observed periods (mean number of days and range) between each participant study encounter.

Among participants with elevated VLs at baseline, 93.2% (82/88) received results (Fig 1). Mean period between enrollment and receipt of results was 60 days (Fig 2). Approximately 90% (78/88) of all eligible participants had a confirmatory VL test. The average number of days between receipt of elevated VL results and collection of confirmatory specimen was 68. Among participants with confirmed elevated VL, mean time from enrollment to second-line treatment initiation was 181 days (range: 125–381). Over half (54%; 27/50) of participants who were eventually switched, initiated second-line therapy on the same day they received confirmation of high VL.

Approximately 7% of participants never received results during the study follow-up period: 89 were terminated from the study prior to receiving results (enrolled ≥6 months without being given results), 6 died, 4 defaulted from care, 2 moved, and 1 was referred immediately for second-line therapy. Participants that were terminated without receiving results were enrolled an average of 195 days (range: 179–322). Four participants did not have VL results because of failed redraw attempts (n = 3) or ineligibility at enrollment (n = 1).

#### Effectiveness

Among participants with a confirmed elevated VL, 92.6% (50/54) initiated second-line therapy. Over 90% (49/54) of participants who were confirmed as eligible for second-line therapy were switched within 365 days of their first elevated VL; over half (31/54) were switched within 180 days. If we assume that the four participants who were switched before confirmatory VLs would not have resuppressed, 91.4% (53/58) of participants reached the primary effectiveness endpoint—initiating second-line therapy within 12 months of enrollment.

## Discussion

DBS for VL monitoring was feasible and effective when implemented by ART providers in district hospitals in Malawi. Greater than 99% of the VL results were available on site, and nearly 80% of participants received their VL results within 3 months of testing. Among the participants with confirmed elevated VL, 92.6% initiated second-line therapy, 91% were switched within one year of their first high VL, and >50% switched to second-line the same day that they received confirmatory VL results.

The time to second-line initiation was considerably faster than has been seen elsewhere in sub-Saharan Africa, where only 62% of patients meeting guideline-dictated failure definitions were switched and a median time between confirmation of failure and treatment switch approached 5 months [24, 30]. There are many potential explanations for the observed differences. Providers involved in our study were aware of study endpoints, whereas retrospective programmatic evaluations in comparator studies are less likely to be subject to that influence.

Nearly one-third (31%) of participants with elevated VLs resuppressed, in-line with previously observed resuppression rates [48–50]. Resuppression suggests that, when coupled with appropriate adherence counseling, VL monitoring can help curtail virological failure. Per national guidelines, providers were instructed to emphasize the importance of adherence for patients with elevated VL [45]. However, we observed substantial inter-clinic variation in rates of resuppression—potentially indicating variable quality of adherence counseling. Alternatively, inter-clinic resuppression variation may be explained by the differences across clinics in time between enrollment and confirmatory testing among participants with elevated VLs. As a component of VL scale-up, Ministry of Health officials should consider monitoring inter-clinic variation in resuppression rates to help identify concordance with guidelines in terms of appropriateness of confirmatory testing as well as needs for additional training in adherence counseling techniques.

We observed surprisingly low virological failure (5.9%) among this previously unmonitored mature ART-patient cohort [14, 24, 26, 27, 29–31]. The lower-than-expected failure rate may be at least partially explained by cohort variability in failure definitions [1, 30, 33]. The low failure rate is unlikely due to inadequate DBS sensitivity, as earlier investigations demonstrate 100% sensitivity of DBS for failure thresholds >5,000 copies/ml, compared to plasma [33]. Our results represent virological failure rates among persons retained in care and thus may underestimate the true rate of failure: 2% of Malawian ART patients default from care each quarter and >18% of patients initiated on ART have been lost to follow-up since 2004, with an additional 10% known to have died [42].

Both age and time on therapy were associated with treatment failure in multivariable models. Younger participants were at increased risk of failure, highlighting the importance of targeting adherence interventions to youth, regardless of how long they are retained on therapy [24, 47, 51]. We only enrolled participants ≥18 years, the majority (>95%) of whom initiated ART in their early or mid-20s and yet were still at increased risk of failure. Expanding the definition of youth to include young adults and tailoring interventions to this group may be an efficient strategy for reducing virological failure. Participants who had been on therapy longer were at increased risk of failing, even after adjusting for clinical signs of failure.

Clinical symptoms were not associated with increased risk of failure, emphasizing shortcomings of relying on clinical staging for predicting virological failure [1, 3, 6]. However, participants enrolled outside of routine eligibility criteria due to suspected clinical failure were significantly more likely to fail than participants meeting routine monitoring eligibility criteria. The difference may be explained by the extent of symptoms: patients enrolled for suspicion of clinical failure were more likely to have multiple concurrent symptoms. Our findings reaffirm the efficiency of monitoring based on suspected clinical failure [1, 52], but demonstrate that nearly 75% of failing patients would be missed if only patients with provider-identified clinical failure received monitoring.

We attempted to have study conditions mimic real-world circumstances, but elements of our evaluation may not be replicated beyond the study setting. Providers were aware of data collection procedures, leading to an unavoidable observer effect. Laboratory turnaround time likely represents an ideal scenario: study coordinators retrieved specimens during site visits when hospital vehicles were not available for specimen transfer, and technicians at the research laboratory had extensive experience with the testing platform. Nonetheless, we observed substantial delays in return of results. Having a “point person” in each hospital or district to facilitate specimen transfer and result follow-up may expedite monitoring activities.

Numerous barriers remain to widespread implementation of DBS for VL monitoring. More than three-fourths of participants who went >90 days without receiving results had at least one interim clinic visit. Laboratory-driven delays (result not available at the time of visit) accounted for some of the “missed” result delivery opportunities. Improved collection and utilization of patient tracing may facilitate improved result delivery turnaround time in the event of laboratory-based processing delays. Patients for whom results are not available at the time of initial visit could be contacted and asked to return for results between regularly scheduled visits—especially relevant for patients with high VLs. However, laboratory delays accounted for only half of the result delivery “misses”, the remaining were due to providers failing to retrieve results and deliver to participants despite being available at the clinic. Improved data management systems at both central laboratories and testing clinics will be critical to improve the result turnaround time. Integrating VL results with existing clinical management by linking results directly to patient records and generating visual flags for clinicians may reduce the frequency with which providers miss delivering available results to patients during clinic visits.

The DBS technology also has some inherent limitations, with poor specificity at lower levels of viremia (<5,000 copies/ml), overestimating VLs by detecting cell-associated HIV DNA and RNA not detected by traditional plasma measurements [33, 35]. Given variation in nucleic acid amplification methods, different VL measurement platforms may be more or less susceptible to these sources of error. Appropriate thresholds for defining virological failure using DBS remains a topic of debate. Nonetheless, high DBS sensitivity makes this monitoring approach an appealing alternative for VL screening programs, likely reducing the number of missed virological failures compared to clinical or immunological monitoring. We did not directly compare DBS performance to clinical and/or immunological failure criteria. However, our assessment of clinical symptoms suggests that clinical monitoring alone would miss a substantial proportion of patients who, according to DBS, were virological failures. Previous work has demonstrated comparability of DBS to plasma in this patient population [33], and although plasma is a more precise means of identifying virological failure, the logistical and financial barriers associated with plasma make it unappealing for the more remote clinical settings explored here.

We have demonstrated that DBS for VL monitoring is both feasible and effective in resource-limited settings. The centralized laboratory testing was efficient and results were successfully distributed back to clinics. Our results help validate the potential for central laboratory testing of DBS to improve access to VL monitoring in more remote settings. Delays in returning results to participants were largely due to inadequate result tracking and provider notification in the existing paper-based and electronic ART management systems. Modifications to these systems will be essential in advance of widespread DBS implementation. We observed remarkable performance in terms of proportion of eligible participants switched to second-line therapy in a timely manner. We also observed a lower-than-expected virological failure rate. At this failure rate, pooling specimens may be a cost-effective testing alternative, although tradeoffs in sensitivity and specificity are important to consider with the dilution effects of pooling, especially at lower failure thresholds [31, 53]. Our findings demonstrate the importance of virological monitoring among patients on ART for extended periods, regardless of clinical symptoms. Important next steps include assessment of resistance among patients who do not resuppress to distinguish between modifiable inadequate adherence and biological failure.
